# Multi-Response Modeling for Bio-Compound Ultrasound-Assisted Extraction (UAE) from Matico (*Piper aduncum* L.) and Chacruna (*Psychotria viridis* Ruiz & Pav.) Leaves Originating in the Peruvian Amazon

**DOI:** 10.3390/molecules30224395

**Published:** 2025-11-13

**Authors:** Raquel Rafael-Saldaña, Roifer Pérez-Vasquez, José Luis Pasquel-Reátegui, Manuel Fernando Coronado-Jorge, Pierre Vidaurre-Rojas, Ángel Cárdenas-García, Keller Sánchez-Dávila, Keneth Reátegui-Del Águila

**Affiliations:** 1 Grupo de Investigación en Ingeniería y Tecnología Agroindustrial, Facultad de Ingeniería Agroindustrial, Universidad Nacional de San Martín (UNSM), Tarapoto 22000, Peru; rrafaels@alumno.unsm.edu.pe (R.R.-S.); rperezv@alumno.unsm.edu.pe (R.P.-V.); mfcoronado@unsm.edu.pe (M.F.C.-J.); 2 Grupo de Investigación Gestión ATEC, Facultad de Ciencias Económicas, Universidad Nacional de San Martín (UNSM), Tarapoto 22200, Peru; pvidaurre@unsm.edu.pe; 3 Facultad de Ingeniería de Sistemas e Informática, Universidad Nacional de San Martín (UNSM), Tarapoto 22201, Peru; acardenasg@unsm.edu.pe; 4 Facultad de Medicina Humana, Universidad Nacional de San Martín (UNSM), Tarapoto 22000, Peru; ksanchezd@unsm.edu.pe; 5 Faculty of Engineering and Environmental Sciences, Universidad Nacional Intercultural de la Amazonia (UNIA), Pucallpa 22200, Peru; kreateguid@unia.edu.pe

**Keywords:** response surface, yield, phenolics, antioxidants, Box–Behnken, medicinal plants

## Abstract

Medicinal plants play an essential role in the food, pharmaceutical, and cosmetic industries due to their ability to prevent and treat diseases. In this study, a three-factor, three-level Box–Behnken experimental design (BBD) with response surface methodology (RSM) was used to maximize the conditions of ultrasound-assisted extraction (UAE) of bioactive compounds from matico and chacruna leaves in terms of total extraction yield (TEY), total phenolic content (TPC) and antioxidant activity (AA) using ABTS and DPPH assays. The effect of methanol concentration (X1: 25%, 50%, and 75%), time (X2: 3, 6, and 9 min), and power (X3: 90, 270, and 450 W) was evaluated as independent variables. The experimental results were fitted to second-order polynomial models, and multiple regression analysis and analysis of variance were used to determine the suitability of the models, using which 3D response surface plots were generated. Considering the multivariable optimization, the best extraction conditions were 73.68% *v*/*v* methanol, 9 min, 269.32 W for matico, and 64.84% *v*/*v* methanol, 3 min, 344.44 W for chacruna. Under these conditions, the maximum value of 18.33 and 20.83% for TEY, 7.16 and 40.86 mg GAE/g dm for TPC, 56.88 and 526.38 µmol TE/g dm for DPPH were predicted for matico and chacruna, respectively. Practical Applications: This research focused on the modeling by response surface methodology (RSM) of Ultrasound-Assisted Extraction of bioactive compounds from matico and chacruna, Peruvian plants used in traditional medicine. The methodologies used allow the maximization of bioactive extraction, which presented a high recovery of phenolics with high antioxidant activity. These results highlight the use of Amazon plants in traditional medicine and their possible use in other industries such as cosmetic or food safety.

## 1. Introduction

Several plant species have a fairly widespread empirical use due to their medicinal properties [1]. Medicinal plants are a more affordable and convenient alternative for treating multiple conditions and are linked to their active constituents [2]. In addition, many drugs currently used were developed from plants used in traditional medicine, so there is great interest in the rediscovery of medicinal plants as a source of possible medicines [3].

The active principles identified in various plant species can be used to formulate medicines, cosmetics, and food products [4]. Although synthetic molecules with biological activity are currently available, consumers prefer products of natural origin because they are perceived as safer, and that is why plants continue to be used for medicinal purposes [5].

*Piper aduncum* L. (matico) is a species native to Peru and is frequently used in traditional medicine [6]; antiviral, antifungal, and antibacterial properties are attributed to it, and it is used for the treatment of nausea, ulcers, hemorrhages, cystitis, respiratory diseases, wounds, and muscle injuries, which is supported by the presence of secondary metabolites found in the phytochemical analysis [7].

*Psychotria viridis* (chacruna) is used to treat fever and is a key component of the famous drink “ayahuasca”. The plant contains N,N-dimethyltryptamine, which, when combined with monoamine oxidase inhibitors, produces psychoactive and hallucinogenic effects [8]. Beyond its traditional use, *Psychotria viridis* has medicinal applications as an antidepressant, anticancer, and antiviral agent [9]. The majority of studies focus on the determination and quantification of N,N-dimethyltryptamine (DMT) [10].

The extraction of natural phenolic compounds has been the subject of interest due to their high antioxidant activity and applicability in the food, medicinal, and pharmaceutical fields [11]. Extraction is the first and most important step in recovering and purifying bioactive compounds from plants [12]. Conventional techniques have disadvantages, such as long times, residual solvents, high costs, low flexibility, and degradation of the compounds of interest, and others [13].

Ultrasound-assisted extraction (UAE) is a novel alternative considered a green technology, with advantages such as short extraction times, low temperature, lower energy, and solvent consumption, in addition to having scalability potential [14]. However, it becomes more efficient due to the phenomenon of acoustic cavitation, which originates from the creation, growth, and implosion of gas bubbles that collapse on the surface of the plant material, releasing high pressure and temperature, thereby facilitating the penetration of the solvent [15].

The response surface methodology (RSM) is a statistical and mathematical technique that allows modeling and optimizing extraction processes of bioactive components [16], through a polynomial equation, a model that relates the effect of the independent variables and their interaction on the response variable [17], and also reduces the number of experiments compared to a complete factorial design [18]. The Box–Behnken design (BBD) is one of the most used in RSM [19,20].

Matico and chacruna are frequently used in traditional Peruvian medicine, but there needs to be more scientific evidence to validate their clinical use. The vast majority of published works deal with extracts obtained by maceration and isolated compounds, especially in the field of chemistry and ethnopharmacology [1,21]. Therefore, the objective of this work was to optimize the ultrasound-assisted extraction (UAE) of matico and chacruna leaves by applying a Box–Behnken design with RSM, in order to evaluate the effect of the extraction conditions: methanol concentration (X1: 25, 50 and 75%), time (X2: 3, 6 and 9 min) and power (X3: 90, 270 and 450 W), on the total extraction yield (TEY), total phenolic content (TPC), and antioxidant activity (AA) by ABTS and DPPH assays.

## 2. Results

### 2.1. Characterization of Matico and Chacruna Leaves

The crushed dried leaves of *Piper modicum* L. and *Psychotria viridis* Ruiz & Pav. were characterized in terms of crude fiber, fat, moisture, total protein, total ash, carbohydrates, and total energy (Table 1).

### 2.2. Using Box–Behnken Experimental Design

The Box–Behnken experimental design was used to evaluate the effect of variables on the extraction process. Table 2 and Table 3 present the experimental and predicted data obtained under the conditions established by the experimental design for matico and chacruna, respectively. Response surface graphs were constructed to visualize the effect of the response variables on the extraction of the compounds of interest. Two variables were varied over different ranges, while the third variable was kept constant [22].

#### 2.2.1. UAE of Matico (*Piper aduncum* L.) Leaves

Methanol concentration had the most significant effect on TEY, TPC, ABTS, and DPPH extraction from matico leaves, followed by time, while power had no effect on the responses (Tables S1–S4).

The effects of the variables on TPC, ABTS, and DPPH were very similar. The high impact of the methanol concentration can be observed in experiments 3 and 4, where the values increased when varying the methanol concentration from 25% to 75%, reaching 2.95 and 7.27 mg GAE/g dm, 41.2 and 92.95 µmol TE/g dm, 15.23 and 57.27 µmol TE/g dm, respectively, for TPC, ABTS, and DPPH (Table 2).

Conversely, the highest TEY values were achieved with low methanol concentrations. For example, in experiments 1 and 2, 20.52% and 14.97% were obtained using 25% and 75% methanol, respectively.

Experiments 10 and 11 illustrate the low influence of time and the absence of the power effect on TPC and ABTS. Similar results were achieved by combining 9 min with 90 W and 3 min with 450 W (4.58 ± 0.11 and 4.54 ± 0.36 mg GAE/g dm; 58.04 ± 4.98 and 59.33 ± 1.89 µmol TE/g dm, for TPC and ABTS, respectively). The effect of time was more significant for the values of TEY and DPPH. This behavior can be observed in experiments 2 and 4 for TEY (14.97% and 18.7%) and in experiments 9 and 10 for DPPH (28.02 and 40.9 µmol TE/g dm), with times of 3 min and 9 min, respectively, under the same methanol concentration and power conditions.

The proposed models have demonstrated their reliability by adequately explaining the experimental data. The lack of adjustment was not significant, affirming their suitability (Table 4). The optimization at the levels studied predicts a maximum value of 23.53% using 40.71% methanol, 9 min, and 173.3 W for TEY. It predicts a result of 7.32 mg GAE/g dm, 93.85 µmol TE/g dm, and 58.41 µmol TE/g dm for TPC, ABTS and DPPH, respectively, under the conditions of 75% methanol, 9 min, and 270 W.

**Figure 1:**
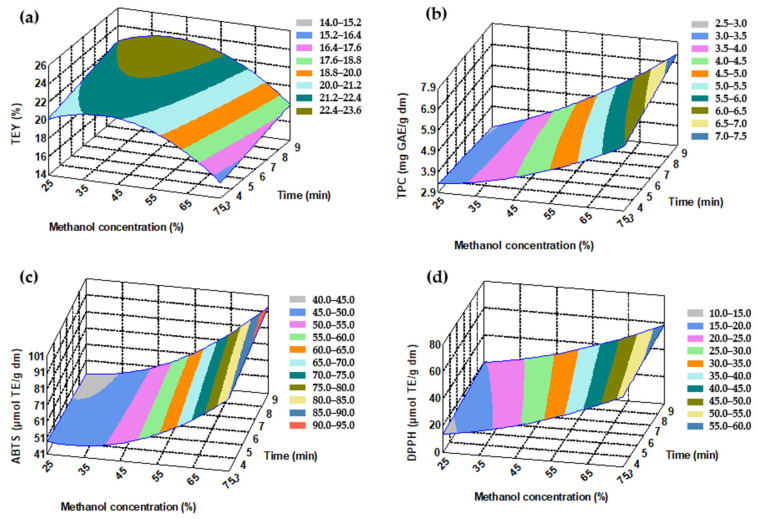
Response surface-matico leaf extracts: (**a**) total extraction yield (TEY), (**b**) total phenolic content (TPC), (**c**) antioxidant activity by ABTS, (**d**) antioxidant activity by DPPH. (The value of the third variable was set at the center point).

Figure 1a shows a small optimized area for TEY, with a methanol concentration between 35% and 45%. Based on this, the matico leaves appear to contain more polar compounds than nonpolar ones. The response surface plots for TPC, ABTS, and DPPH did not display optimal regions under the applied conditions (Figure 1b–d). These results suggest that the extraction process may be further improved to obtain extracts with higher phenolic content and antioxidant activity by studying methanol concentrations above 75% and extraction times longer than 9 min.

#### 2.2.2. UAE of Chacruna (*Psychotria viridis* Ruiz & Pav.) Leaves

In the UAE of bioactive compounds from chacruna leaves, the impact of the variables was less noticeable than in the UAE of matico leaves; even for DPPH, the ANOVA showed that none of the terms were significant (Table S8). Significant and non-significant terms are presented in Tables S5–S8.

The significant effect of methanol concentration can be seen in experiments 1 and 2, where the values increased by varying the methanol concentration from 25% to 75% while keeping the other variables constant (from 17.3% to 21.2%, from 30.17 to 45.73 mg GAE/g dm, from 562.95 to 939.72 µmol TE/g dm, and from 365.51 to 547.55 µmol TE/g dm for TEY, TPC, ABTS, and DPPH, respectively).

Power increased the concentration of bioactive compounds in the UAE of chacruna leaves. Experiments 6 and 8 (Table 3) show the influence of this variable. When the power was increased from 90 W to 450 W, the results for TEY, TPC, ABTS, and DPPH also rose from 9.43% to 14.84%, 20.75 to 27.67 mg GAE/g dm, 422.41 to 534.68 µmol TE/g dm, and 276.08 to 418.65 µmol TE/g dm, respectively.

The content of bioactive compounds decreased with increasing time, as seen in experiments 2 and 4 (Table 3). Increasing the time from 3 to 9 min under the same conditions of methanol concentration and power significantly reduced the values achieved (21.2% to 17.86%, 45.73 to 31.83 mg GAE/g dm, 939.72 to 591.54 µmol TE/g dm, and 547.55 to 404.76 µmol TE/g dm for the values of TEY, TPC, ABTS, and DPPH, respectively).

The R^2^ values of the models proposed for UAE of chacruna leaves were between 76.08% and 82.33% (Table 5), which were lower than those obtained for matico. The lack of fit for the TEY, TPC, and DPPH was not significant, indicating that the models adequately described the experimental data. However, the lack of fit for the ABTS response was significant (*p*-value = 0.0068), indicating that the proposed model did not adequately represent the data and, therefore, should not be used for prediction purposes. The optimization at the levels studied predicted a maximum value of 21.28% with 53.93% methanol, 3 min, and 321.06 W for TEY; 41.34 mg GAE/g dm with 70.82% methanol, 3 min, and 313.48 W for TPC; and 539.03 µmol TE/g dm under the conditions of 75% methanol, 3 min, and 432.66 W for DPPH.

**Figure 2:**
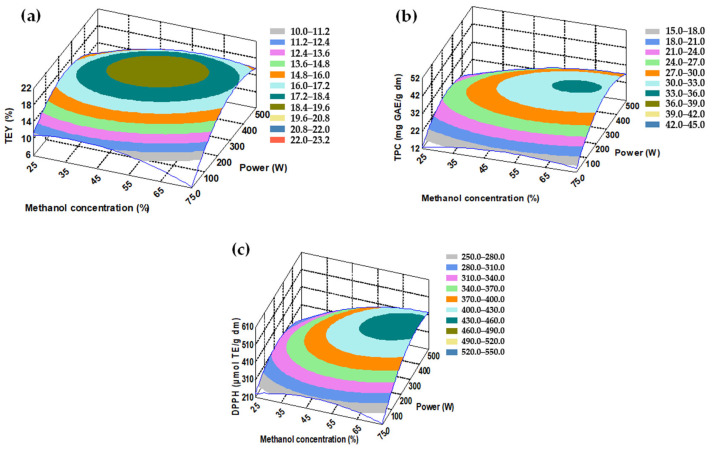
Response surface-chacruna leaf extracts: (**a**) total extraction yield (TEY), (**b**) total phenol content (TPC), (**c**) antioxidant activity by DPPH assay. (The value of the third variable was set at the center point).

When analyzing the response surfaces (Figure 2), small regions optimized at the studied levels for methanol concentration and power (Figure 2a–c) were observed, and their values coincided with those mentioned previously. There were no optimized regions in the graphs where time was involved, which follows a trend suggesting that studying times of less than 3 min could further improve the extraction process. For both matico and chacruna, the response surface graphs of antioxidants were very similar to those of TPC, which is explained by the strong correlation between TPC and the AA of the extracts, as explained in Section 2.3.

#### 2.2.3. Simultaneous Optimization of All Responses

To evaluate the extraction conditions that optimize all the response variables for matico and chacruna (using the models presented in Table 4 and Table 5), an optimization study was conducted using the “Optimize Responses” option of Statgraphics Centurion software (18.1.13). The purpose was to estimate the extraction conditions that would be expected to yield high TEY, TPC, ABTS, and DPPH values. Table 6 shows the maximum values predicted by individual optimization and those that would theoretically be obtained under multivariable optimization.

For the simultaneous optimization of the chacruna data, the ABTS response was excluded because, as previously noted, its model showed a significant lack of fit, making it unsuitable for prediction. Therefore, the optimization results (Table 6) represent theoretical values corresponding to the predicted optimal conditions, which could be experimentally validated to confirm the reliability of the models.

### 2.3. Correlation Between TPC and AA of Matico and Chacruna Leaf Extracts

**Figure 3:**
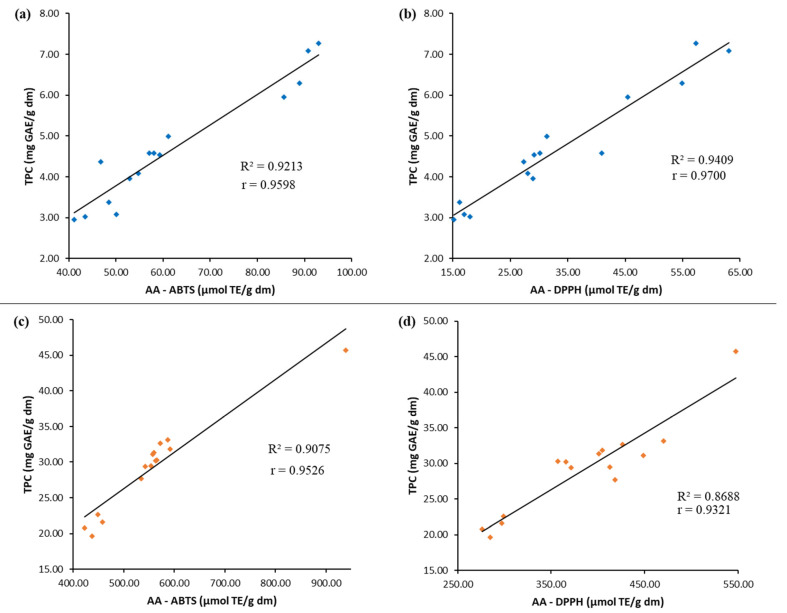
Correlation between the antioxidant activity (AA) and the total phenolic content (TPC) of the extracts obtained by UAE under different conditions. Matico leaf extracts (**a**,**b**). Chacruna leaf extracts (**c**,**d**).

Figure 3 shows that the Pearson correlation coefficients (r) between AA (measured by ABTS and DPPH) and TPC ranged from 0.9321 to 0.9700. Therefore, we can affirm that phenolic compounds are primarily responsible for the antioxidant activity in the extracts of matico and chacruna leaves.

## 3. Discussion

Since no reports had been found that allowed for comparison of the characterization of matico and chacruna leaves, a search was conducted on the proximal composition of other species belonging to the genera *Piper* and *Psychotria*. The dried leaves of *Piper guineense* showed 18.19% crude fiber, 2.45% fat, 11.55% moisture, 11.32% protein, 14.25% ash, and 42.23% carbohydrates [23]. Meanwhile, the dried leaves of *Psychotria* sp. contained 3.40% crude fiber, 8.55% fat, 12.87% moisture, 27.32% protein, 7.06% ash, and 40.80% carbohydrates [24]. Some of the mentioned data were relatively similar; however, the variability could be explained by the influence of factors such as species, soil, climatic conditions, altitude, and collection time [25]. These factors caused significant qualitative and quantitative changes in the phytochemical profile of these plants [6,21].

Most studies on matico and chacruna refer to phytochemical analyses. Matico leaf extracts have been found to contain a high concentration of flavonoids, followed by a moderate concentration of phenols, alkaloids, and terpenoids [26]. Certain phenols and flavonoids, including gallic acid, catechin, chlorogenic acid, caffeic acid, ellagic acid, rutin, quercetin, luteolin, and apigenin, have been detected in chacruna extracts [27]. Various factors influenced the extraction of active compounds, such as the solvent, solute:solvent ratio, time, temperature, ultrasonic power, and particle size. The influence of these parameters varied depending on the plant and the responses analyzed [28].

When working with response surface modeling, a high R^2^ and a non-significant lack of fit (*p* > 0.05) are indicators that the quadratic model is reliable and that the variation between samples is due only to the factors selected by the model and pure error [29].

In the UAE of matico (*Piper aduncum* L.) leaves, the lack of significance of the power variable is likely due to the fact that the particles were very small (powdered) and did not hinder mass transfer [25]. In addition, the methanol/water mixture destroys cell membranes, allowing the release and stabilization of certain phenolic subgroups. Likewise, methanol mitigates the degradation of phenols by inhibiting the activity of polyphenol oxidases, which are abundant in plants [30]. The positive effect of time was likely due to greater contact between the solvent and the plant material, which facilitated the diffusion of the tissue components into the solvent [31]. This may be related to an increase in the formation of microbubbles, which intensify cellular damage by facilitating the release of phytochemicals [32].

Only for TPC, ANOVA showed a significant interaction between methanol concentration and extraction time (Table S2). According to the fitted model (Table 4), this interaction was positive, indicating that higher methanol concentrations combined with longer extraction times resulted in greater recovery of phenolic compounds. This behavior may be attributed to the enhanced solubility and diffusion of phenolics when sufficient extraction time is provided.

Other authors have reported results for TPC and AA of matico leaf extracts. However, because the results were expressed in various ways, comparing them with those obtained in this study is difficult. Arroyo et al. [33] reported a TPC of around 16 mg GAE/mL for the methanolic fraction of an extract; Yarleque-Chocas et al. [1] reported a value of 21.12 mg GAE/g sample using 96% ethanol through maceration for seven days; Luna-Fox et al. [34] reported values of 2.24 g GAE/100 g dm in an aqueous extract using low-power ultrasound, and antioxidant activity measured by the ABTS assay of 86.4 mg TE/100 g dm, respectively.

It was not convenient to reparameterize the polynomial models for the chacruna leaf data. Sometimes, when only the significant terms are considered, there can be a high error between the experimental and predicted data; therefore, it is more appropriate to use complete polynomial models [20].

In the UAE of chacruna (*Psychotria viridis* Ruiz & Pav.) leaves, the negative impact of the quadratic effect of power on TEY, TPC, and DPPH indicates that extraction improves as power increases up to a certain point and then begins to decrease. This could be attributed to the possible degradation of compounds caused by the ultrasonic power supplied, which raises the system’s temperature due to the magnitude of the cavitation generated [35].

The negative impact of time on TEY, TPC, and DPPH may result from the thermal degradation of the compounds present in chacruna leaves [36]. This degradation is caused by the combination of high power and prolonged times, which triggers high temperatures and pressures [31]. Prolonged exposure to ultrasound can cause adverse effects, such as reduced levels of phenolic compounds due to forming free radicals [36,37]. This leads to chemical decomposition, negatively affecting free radical scavenging activity [38]. It has also been reported that prolonged extraction periods reduce phenol levels due to oxidation [36].

The lower R^2^ values in the models obtained for chacruna compared with those achieved for matico could be due to the variability of the compounds, including phenolics since these molecules have various polarities and sizes, ranging from simple phenols to tannins [19].

To our knowledge, the content of phenolic compounds and the antioxidant activity of chacruna leaves have not yet been investigated. For comparative purposes, a study was found on extracts from *Psychotria nilgiriensis* leaves, where the authors reported 66.11 mg GAE/g extract with methanol as a solvent; the antioxidant activity was 833.3 µg/mL by DPPH and 1277.43 µM Trolox equivalent/g extract by ABTS [39].

In general, the highest TEY values for matico and chacruna were achieved with 50% methanol, whereas the phenolic compounds were extracted in greater amounts with 75% methanol concentrations (Table 2 and Table 3). Mixtures of solvents with water increase diffusivity and improve the extraction rate [40] A higher water content also enhances the extraction of certain contaminant solutes, affecting the concentration of phenolic compounds [41]. In other words, decreasing the concentration of methanol will result in extracts with a high yield (due to the presence of contaminating materials) but with lower antioxidant activity [42].

Both species studied show variability in their content of bioactive compounds compared to other studies. Factors such as species, soil, climatic conditions, altitude, and collection time cause these differences [25]. It is also difficult to compare with other reports because the results are not expressed uniformly.

The solvent concentration is one of the most significant variables in the UAE [42]. This was reflected in our results, where the methanol concentration was the variable that most affected the extraction of bioactive compounds from matico and chacruna leaves. The mixture of methanol and water decreases viscosity, facilitating cavitation [38]. The maximized conditions (Table 6) coincide with those reported in other studies. For example, Yerena-Prieto et al. [37] observed that the methanol concentration is the most influential variable in extracting bioactives from moringa leaves.

Several studies have found a proportional relationship between the increase in solvent concentration up to a certain point (solvent/water mixtures) and the content of phenolic compounds [17,19,20]. However, it is necessary to carry out studies to confirm which solvent is the most suitable since the solubility of TPC cannot be explained solely by its polarity, other factors also influence it, such as stereochemistry, intermolecular forces with the solvent [43], and the nature of the plant itself [25].

The bioactive compounds of the matico were not affected by the ultrasonic power or the increase in time, indicating good thermal stability. At the same time, these variables degraded the phytochemicals of the chacruna, indicating that they would be more sensitive to temperature. This behavior can be explained by the thermostability of phenolic compounds, which depends on their chemical nature [44].

The ABTS values were higher than those of DPPH (Table 2 and Table 3), possibly due to the presence of various phenolic compounds with different antioxidant capacities and the varying mechanisms of action of the antioxidant assays [45].

Consistent with our findings several studies have pointed out an important dependence between the content of phenolic compounds and antioxidant activity [20,42]. It is essential to know if the phenolic compounds in the extracts exhibit antioxidant activity, because this characteristic is linked to health benefits and is highly sought after in various industries [20].

## 4. Materials and Methods

### 4.1. Material

Fresh leaves of *Piper aduncum* L. (matico) and *Psychotria viridis* Ruiz & Pav. (chacruna) were collected from an experimental center located in the cities of Rioja and Tarapoto, respectively (San Martín, Peru), in September 2022. In both cases, the methodology proposed by Farahmandfar et al. [40] was followed. The leaves were separated, cleaned, and dried under shade at room temperature for one week. The samples were then ground using a blade mill (OSTER) to homogenize the material. The milled leaves were placed in airtight glass jars and refrigerated at 2 °C until further analysis.

ABTS (2,2′-azino-bis-(3-ethylbenzothiazoline)-6-sulfonic acid), DPPH (1,1-diphenyl-2-picryl- hydrazyl), potassium persulfate (CAS 7727-21-1), anhydrous gallic acid (CAS 149-91-7), and Trolox were purchased from Merck KGaA (Darmstadt, Germany). Sodium carbonate (CAS 144-55-8) and Folin–Ciocalteu reagent were purchased from Tawa (Lima, Peru). Methanol (CAS 67-56-1, purity 99.8%) was purchased from Grupo Química (Lima, Peru). Distillation equipment was used for the water (2008, GFL, Burgwedel, Germany).

### 4.2. Characterization of Raw Material

The dried and crushed leaves were characterized in terms of crude fiber using the Peruvian Technical Standard (NTP 205.003:1980) [46], and fat, moisture, protein, and ash were determined by methods 930.09, 930.04, 978.04 (A), and 930.05, respectively, according to AOAC (2019) [47]. Carbohydrates were estimated by difference, subtracting the sum of moisture, protein, fat, and ash from 100 g of sample, and total energy was calculated using the Atwater factors, assigning 4 kcal/g for protein, 4 kcal/g for carbohydrates, and 9 kcal/g for fat [48].

### 4.3. Ultrasound-Assisted Extraction (UAE)

The extraction was performed using an ultrasonic probe equipment (900 W power, 12.5 mm Ø, frequency 20 kHz, Biobase, Jinan, China). 3 g of sample were mixed with 50 mL of the extracting solvent (methanol/water in different concentrations). After UAE, the extract was centrifuged (EPPENDORF, 5702, Hamburg, Germany) at 3500 rpm for 5 min. The supernatant was collected and stored in amber bottles at 2 °C until further characterization.

### 4.4. Box–Behnken Experimental Design

The UAE of bioactive compounds from matico and chacruna leaves was carried out according to a Box–Behnken experimental design. The experimental conditions were generated with Statgraphics Centurion software, giving 15 experiments (3 center points and 12 combinations of independent variables, Table 2 and Table 3). The independent parameters were the methanol concentration (X_1_), extraction time (X_2_) and power (X_3_). The experimental data was fitted with a second-order polynomial equation (Equation (1)) to maximize the response variables (TEY, TPC, ABTS, and DPPH).

(1) $$Y=\beta_{0}+\sum \beta_{i}x_{i}+\sum \beta_{ii}x_{ii}^{2}+\sum \beta_{ij}x_{i}x_{j}+\varepsilon$$

where Y is the response variable, $\beta_{0}$ is an independent parameter, $\beta_{i}$ is the linear effect of the input factor $x_{i}$, $\beta_{ii}$ is the quadratic effect of the input factor $x_{i}$, and $\beta_{ij}$ represents the linear interaction effect between the input factor input $x_{i}$ and $x_{j}$, ε symbolizes the error. The suitability of the models was evaluated through the multiple regression coefficients (R^2^) and lack of fit [17].

New models were generated for the UAE of matico leaves by eliminating coefficients that were not significant (*p* > 0.05). Meanwhile, for chacruna leaves, the models were analyzed including all the terms, because when attempting to reparameterize, very low R^2^ and some significant lack of fit were obtained (*p* < 0.05).

### 4.5. Extract Characterization

#### 4.5.1. Total Extraction Yield (TEY)

To determine the TEY, the methodology proposed by Vélez-Erazo et al. [49] was followed. 3 mL of extract were placed in a Petri dish and dried in a conventional oven (Venticell 111-ECO line, BMT medical, Kassel, Germany) at 105° C, evaporating the solvent until a constant weight was obtained. The results were calculated with Equation (2).

(2) $$TEY\;\left(\%\right)=\frac{W_{1}\times 100}{W_{2}}$$

where *W*_1_ = weight of the dry extract, and *W*_2_ = initial weight of the sample. The weights were determined on an analytical balance (Pioneer TM PX224, OHAUS, Mexico City, Mexico).

#### 4.5.2. Total Phenolic Content (TPC)

The total phenolic content was determined using the Folin–Ciocalteu spectrophotometric method described by Singleton et al. [50], with some modifications. In brief, 200 μL of extract, 200 μL of diluted Folin–Ciocalteu reagent (1:2), 400 μL of sodium carbonate (10% *w*/*v*), and 3200 μL of distilled water were mixed, stirred for 5 s, and incubated for 30 min at 25 °C in the dark. The absorbance was measured at 765 nm in a spectrophotometer (S-220 UV/VIS, BOECO, Hamburg, Germany). The calibration curve was prepared with a standard solution of gallic acid (0–0.12 mg/mL). Equation (3) was used to calculate the results [51].

(3) $$Tpc=\frac{c\times V}{m}$$

where *c* = concentration of the sample in the calibration curve, *V* = volume of the solvent used, and *m* = sample weight. The results were expressed as mg of gallic acid equivalents (GAE) per gram of dry matter (dm).

#### 4.5.3. Antioxidant Activity-ABTS (Ammonium 2,2′-Azino-bis-(3-ethyl benzothiazolin-6-sulfonate))

The antioxidant activity by ABTS assay was determined through the spectrophotometric method described by Re et al. [52], with certain modifications. 5 mL of ABTS aqueous solution (7 mM) were reacted with 5 mL of potassium persulfate solution (2.45 mM) and kept in the dark for 16 h. Then, the prepared solution was diluted in ethanol in a 1:90 ratio and adjusted to an absorbance of 0.700 ± 0.02 at 734 nm. 20 μL of extract and 2 mL of ABTS^+^ solution were mixed, incubated for 6 min, and then the absorbance was measured at 734 nm. The calibration curve was constructed with a standard Trolox solution (500–2000 μM). The antioxidant activity of the extracts was expressed as μmol Trolox equivalent (μmol TE)/g dm.

#### 4.5.4. Antioxidant Activity-DPPH (1,1-Diphenyl-2-picryl-hydrazil)

Antioxidant activity by DPPH assay was evaluated according to Brand-Williams et al. [53], with some adaptations. The calibration curve was constructed with a standard Trolox solution (20–200 µM). First, the DPPH solution was calibrated to an absorbance of 0.800 ± 0.02 at 515 nm. Then, 500 μL of extract, 3 mL of ethanol, and 300 μL of DPPH solution were added, shaken for 5 s and incubated in the dark for 45 min. The percentage of radical scavenging activity (*RSA*) was calculated using Equation (4).

(4) $$\left(\%\right)RSA=\left(\frac{{Abs}_{control}-{Abs}_{sample}}{{Abs}_{control}}\right)\times 100$$

where ${Abs}_{control}\;$= initial absorbance of the methanolic DPPH solution ${Abs}_{muestra}\;$= absorbance of the reaction mixture (DPPH + sample). The AA of the extracts was expressed as μmol Trolox equivalent (μmol TE)/g dm.

### 4.6. Statistical Analysis

Three experimental repetitions were carried out for each treatment, expressed as average ± standard deviation. Statgraphics Centurion software (trial version 18.1.13, Stat Point Technologies, Inc., Richmond, VA, USA) was used for statistical analysis of the results by RSM. The independent variables’ linear, quadratic, and interaction effects on the responses were established with the analysis of variance (ANOVA) with a significance level of 5%. The adequacy and precision of the models were evaluated using the multiple regression coefficient (R^2^) and lack of fit. To obtain the values of methanol concentration (X_1_), time (X_2_), and power (X_3_) that allow for maximizing the results, the “Optimize Response” software option was used. This was done for each response variable separately, followed by simultaneous optimization. Pearson’s correlation coefficient was used to determine the linear correlation between TPC and AA with Microsoft Office Excel 2019 software.

## 5. Conclusions

In the extracts of matico leaves, methanol concentration was the most significant variable, followed by time, while power had no effect. Likewise, the reparametrized quadratic models adequately adjusted the experimental results and could be used to make predictions. The response surface graphs suggest that the extraction process of TPC and antioxidants could be improved with methanol concentrations greater than 75% and extraction times longer than 9 min. The multiresponse analysis at the levels studied, indicated that under the conditions of 73.68% methanol, 9 min, and 269.32 W, up to 18.33%, 7.16 mg GAE/g dm, 91.27 µmol TE/g dm and 56.88 µmol TE/g dm of TEY, TPC, ABTS, and DPPH would be obtained, respectively.

Power, methanol concentration, and time influenced the UAE process of chacruna leaves. The quadratic models were not reparameterized, resulting in lower R^2^ values than those for matico. However, the lack of fit was not significant, demonstrating the models’ reliability (except for the polynomial for the ABTS response), and the ANOVA of DPPH did not show significant terms (*p*-value < 0.05); therefore, other variables, such as the solute/solvent ratio, applying pre-treatments, other solvents, and even different methodologies or experimental designs, should be studied. The response surface graphs also show the trend that extraction could be improved with times of less than 3 min. The optimization conditions in the multi-response analysis at the levels studied were 64.84% methanol, 3 min, 344.44 W, with the model predicting maximum values of TEY, TPC and DPPH (20.83%, 40.86 mg GAE/g dm, and 526.38 µmol TE/g dm, respectively), excluding the ABTS model due to its significant lack of fit.

A strong correlation was observed between the antioxidant activity of the matico and chacruna extracts and their total phenolic content. Ultrasound-assisted extraction can be used to obtain natural antioxidants from matico and chacruna leaves, which could help develop new products in various fields of industry. In future studies, the methodological framework proposed in this work could be applied using environmentally friendly solvents, such as ethanol–water systems, to enhance the sustainability and industrial applicability of the optimized UAE process. The data obtained could serve as a basis to support the medicinal use of these plants; however, more research is required to isolate and characterize the phytochemicals responsible for their medicinal properties.

## Figures and Tables

**Table 1:** Proximal composition of matico (*Piper aducum* L.) and chacruna (*Psychotria viridis* Ruiz & Pav.) leaves.

	*Piper aducum* L.	*Psychotria viridis* Ruiz & Pav.
Moisture (%)	6.7 ± 0.11	10.5 ± 0.02
Total protein (%)	20.8 ± 0.00	10.3 ± 0.06
Fat (%)	4.9 ± 0.00	0.7 ± 0.00
Total ash (%)	14.2 ± 0.01	10.4 ± 0.01
Carbohydrate (%)	53.4	68.1
Crude fiber (%)	10.7 ± 0.05	16.2 ± 0.04
Total Energy (kcal)	340.9	319.9

Note: The carbohydrate content was determined by difference (fats, moisture, proteins, and ashes).

**Table 2:** Box–Behnken design matrix and experimental results obtained in the ultrasound-assisted extraction of bioactive compounds from matico.

E	Independent Variables			Chemical Characterization of the Extracts							
	X_1_ (%)	X_2_ (min)	X_3_ (W)	TEY	A	TPC	A	ABTS	A	DPPH	A
01	−1 (25)	−1 (3)	0 (270)	20.52 ± 1.06	20.15	3.38 ± 0.21	3.32	48.49 ± 1.94	48.84	16.19 ± 0.5	13.35
02	+1 (75)	−1 (3)	0 (270)	14.97 ± 0.34	15.72	5.95 ± 0.59	5.98	85.7 ± 4.7	85.33	45.45 ± 0.64	51.92
03	−1 (25)	+1 (9)	0 (270)	21.58 ± 0.41	22.35	2.95 ± 0.16	2.90	41.2 ± 3.48	42.82	15.23 ± 1.36	19.84
04	+1 (75)	+1 (9)	0 (270)	18.7 ± 0.16	17.91	7.27 ± 0.18	7.32	92.95 ± 2.43	93.85	57.27 ± 2.71	58.41
05	−1 (25)	0 (6)	−1 (90)	21.41 ± 0.3	21.25	3.08 ± 0.31	3.11	50.12 ± 4.91	45.83	16.97 ± 0.23	16.60
06	+1 (75)	0 (6)	−1 (90)	15.67 ± 0.37	16.81	6.3 ± 0.1	6.65	88.91 ± 7.36	89.59	54.86 ± 4.95	55.17
07	−1 (25)	0 (6)	+1 (450)	21.49 ± 0.96	21.25	3.03 ± 0.28	3.11	43.51 ± 0.51	45.83	17.99 ± 0.64	16.60
08	+1 (75)	0 (6)	+1 (450)	17.91 ± 1.06	16.81	7.08 ± 0.21	6.65	90.81 ± 5.39	89.59	63.09 ± 2.12	55.17
09	0 (50)	−1 (3)	−1 (90)	20.41 ± 1.58	20.92	4.08 ± 0.37	4.21	54.79 ± 3.16	55.09	28.02 ± 0.56	27.62
10	0 (50)	+1 (9)	−1 (90)	21.62 ± 0.67	23.11	4.58 ± 0.11	4.67	58.04 ± 4.98	56.34	40.9 ± 3.19	34.11
11	0 (50)	−1 (3)	+1 (450)	21.84 ± 0.28	20.92	4.54 ± 0.36	4.21	59.33 ± 1.89	55.09	29.18 ± 1.07	27.62
12	0 (50)	+1 (9)	+1 (450)	24.61 ± 0.41	23.11	4.99 ± 0.43	4.67	61.13 ± 1.91	56.34	31.4 ± 2.8	34.11
13	0 (50)	0 (6)	0 (270)	22.45 ± 0.48	22.02	4.59 ± 0.09	4.44	57.07 ± 4.5	55.72	30.21 ± 0.83	30.86
14	0 (50)	0 (6)	0 (270)	21.12 ± 0.54	22.02	3.96 ± 0.35	4.44	52.86 ± 4.27	55.72	28.94 ± 0.17	30.86
15	0 (50)	0 (6)	0 (270)	22.08 ± 0.16	22.02	4.37 ± 0.08	4.44	46.79 ± 4.26	55.72	27.38 ± 1.81	30.86

Data are shown as means ± standard deviations (*n* = 3). E = experiment; X_1_ = methanol concentration; X_2_ = time; X_3_ = power; A = predicted; TEY = total extraction yield (%); TPC = total phenolic content (mg GAE/g dm); ABTS = antioxidant activity by ABTS assay (µmol TE/g dm); DPPH = antioxidant activity by DPPH assay (µmol TE/g dm).

**Table 3:** Box–Behnken design matrix and experimental results obtained in the ultrasound-assisted extraction of bioactive compounds from chacruna.

E	Independent Variables			Chemical Characterization of the Extracts							
	X_1_ (%)	X_2_ (min)	X_3_ (W)	TEY	A	TPC	A	ABTS	A	DPPH	A
01	−1 (25)	−1 (3)	0 (270)	17.3 ± 0.4	18.15	30.17 ± 1.22	29.65	562.95 ± 22.39	560.78	365.51 ± 33.98	362.11
02	+1 (75)	−1 (3)	0 (270)	21.2 ± 0.35	18.86	45.73 ± 1.03	40.80	939.72 ± 33.85	822.93	547.55 ± 46.16	500.27
03	−1 (25)	+1 (9)	0 (270)	15.39 ± 0.82	17.73	22.6 ± 1.13	27.53	448.82 ± 3.77	565.61	298.85 ± 7.42	346.13
04	+1 (75)	+1 (9)	0 (270)	17.86 ± 0.42	17.01	31.83 ± 1.04	32.35	591.54 ± 4.56	593.72	404.76 ± 22.14	408.16
05	−1 (25)	0 (6)	−1 (90)	15.8 ± 0.71	14.19	21.6 ± 0.83	19.57	457.95 ± 9.44	409.71	297.24 ± 9.11	283.38
06	+1 (75)	0 (6)	−1 (90)	9.43 ± 0.18	11.01	20.75 ± 1.16	23.13	422.41 ± 19.92	488.79	276.08 ± 23.97	306.10
07	−1 (25)	0 (6)	+1 (450)	14.86 ± 0.11	13.28	19.66 ± 1.69	17.28	438.11 ± 31.37	371.73	285.05 ± 7.4	255.04
08	+1 (75)	0 (6)	+1 (450)	14.84 ± 0.31	16.45	27.67 ± 1.31	29.70	534.68 ± 3.94	582.92	418.65 ± 23.27	432.51
09	0 (50)	−1 (3)	−1 (90)	16.21 ± 0.45	16.97	29.41 ± 0.65	31.96	543.02 ± 16.33	593.43	371.41 ± 38.09	388.67
10	0 (50)	+1 (9)	−1 (90)	16.96 ± 0.57	16.23	29.48 ± 0.56	26.58	554.51 ± 9.94	485.96	412.7 ± 35.48	379.29
11	0 (50)	−1 (3)	+1 (450)	18.88 ± 0.43	19.62	31.09 ± 0.37	33.99	557.67 ± 10.29	626.22	448.96 ± 41.01	482.37
12	0 (50)	+1 (9)	+1 (450)	18.87 ± 1.15	18.11	31.36 ± 0.98	28.81	559.73 ± 14.73	509.32	400.92 ± 8.15	383.66
13	0 (50)	0 (6)	0 (270)	20.14 ± 0.03	19.06	33.14 ± 0.99	32.01	587.78 ± 17.88	575.36	470.4 ± 37.84	418.00
14	0 (50)	0 (6)	0 (270)	18.66 ± 0.81	19.06	32.61 ± 1.63	32.01	572.25 ± 15.76	575.36	426.59 ± 29.53	418.00
15	0 (50)	0 (6)	0 (270)	18.39 ± 0.76	19.06	30.29 ± 0.75	32.01	566.05 ± 18.12	575.36	357 ± 22.33	418.00

Data are shown as means ± standard deviations (*n* = 3). E = experiment; X_1_ = methanol concentration; X_2_ = time; X_3_ = power; A = predicted; TEY = total extraction yield (%); TPC = total phenolic content (mg GAE/g dm); ABTS = antioxidant activity by ABTS assay (µmol TE/g dm); DPPH = antioxidant activity by DPPH assay (µmol TE/g dm).

**Table 4:** Models for total extraction yield, total phenolic content and antioxidant activity (ABTS and DPPH) of matico leaves using UAE.

Response	Reparametrized Models	R^2^ (%)
TEY	$Y=12.31+0.3892X_{1}+0.3654X_{2}-0.00478{X_{1}}^{2}$	88.15
TPC	$Y=3.937-0.03391X_{1}-0.215X_{2}+0.0006971{X_{1}}^{2}+0.005833X_{1}X_{2}$	97.05
ABTS	$Y=73.22-1.335X_{1}-2.215X_{2}+0.01919{X_{1}}^{2}+0.04847X_{1}X_{2}$	96.45
DPPH	$Y=5.88-0.0317X_{1}+1.08X_{2}+0.00803{X_{1}}^{2}$	93.76

**Table 5:** Models for total extraction yield, total phenolic content and antioxidant activity (ABTS and DPPH) of chacruna leaves using UAE.

Response	Non-Reparametrized Models	R^2^ (%)
TEY	$Y=10.53+0.3431X_{1}-1.769X_{2}+0.03691X_{3}-0.004099{X_{1}}^{2}-0.004767X_{1}X_{2}+0.0003528X_{1}X_{3}+0.1595{X_{2}}^{2}-0.0003519X_{2}X_{3}-0.00008547{X_{3}}^{2}$	76.08
TPC	$Y=17.13+0.7411X_{1}-5.507X_{2}+0.07944X_{3}-0.005877{X_{1}}^{2}-0.0211X_{1}X_{2}+0.0004922X_{1}X_{3}+0.4713{X_{2}}^{2}+0.00009259X_{2}X_{3}-0.0001827{X_{3}}^{2}$	82.33
ABTS	$Y=402.55+8.006X_{1}-79.07X_{2}+1.355X_{3}-0.02404{X_{1}}^{2}-0.7802X_{1}X_{2}+0.007339X_{1}X_{3}+8.38{X_{2}}^{2}-0.004366X_{2}X_{3}-0.002995{X_{3}}^{2}$	72.72
DPPH	$Y=146.58+9.449X_{1}-35.43X_{2}+0.7412X_{3}-0.08246{X_{1}}^{2}-0.2538X_{1}X_{2}+0.008598X_{1}X_{3}+4.19{X_{2}}^{2}-0.04136X_{2}X_{3}-0.001457{X_{3}}^{2}$	80.04

**Table 6:** Predicted values of the responses obtained under optimal extraction conditions.

Response	Matico (*Piper aducum* L.) Leaves			Chacruna (*Psychotria viridis* Ruiz & Pav.) Leaves		
	Predicted Optimal Value	Predicted Value by Simultaneous Optimization *	A%	Predicted Optimal Value	Predicted Value by Simultaneous Optimization **	A%
TEY	23.53	18.33	77.92	21.28	20.83	97.84
TPC	7.32	7.16	97.76	41.34	40.86	98.84
ABTS ^†^	93.85	91.27	97.25	-	-	-
DPPH	58.41	56.88	97.37	539.03	526.38	97.65

(A%) = Percentage achieved by simultaneous optimization over the expected optimal value; TEY (%); TPC (mg GAE/g dm); ABTS and DPPH (µmol TE/g dm). * 73.68% methanol, 9 min, 269.32 W. ** 64.84% methanol, 3 min, 344.44 W. ^†^ ABTS model for chacruna showed a significant lack of fit and was excluded from optimization.

## Data Availability

The original contributions presented in this study are included in the article/Supplementary Materials. Further inquiries can be directed to the corresponding author.

## Supplementary Materials

The following supporting information can be downloaded at: https://www.mdpi.com/article/10.3390/molecules30224395/s1, Table S1. Analysis of Variance for TEY (%) of Matico. Table S2. Analysis of variance for TPC (mg GAE/g dm) of Matico. Table S3. Analysis of variance for ABTS (μmol TE/g dm) of Matico. Table S4. Analysis of variance for DPPH (μmol TE/g dm) of Matico. Table S5. Analysis of variance for TEY (%) of Chacruna. Table S6. Analysis of variance for TPC (mg GAE/g dm) of Chacruna. Table S7. Analysis of variance for ABTS (μmol TE/g dm) of Chacruna. Table S8. Analysis of variance for DPPH (μmol TE/g dm) of Chacruna.
