# Supplementary material for: Multi-Response Modeling for Bio-Compound Ultrasound-Assisted Extraction (UAE) from Matico (Piper aduncum L.) and Chacruna (Psychotria viridis Ruiz & Pav.) Leaves Originating in the Peruvian Amazon
Source: Molecules. 2025 Nov 13;30(22):4395. doi: 10.3390/molecules30224395 (PMC12655001; doi:10.3390/molecules30224395)
Supplement: Supplementary file 1 [file molecules-30-04395-s001.zip › molecules-3941788-supplementary.pdf]

Supplementary Material

ANOVA - Box Behnken for the Matico experimental data (Reparameterized)

Table S1. Analysis of Variance for TEY (%) of Matico

Source	*Sum of Squares*	*DF*	*Mean Square*	*F-Value*	*P-Value*
A: Methanol concentration (%)	39.38	1	39.38	26.41	0.0021
B:Time (min)	9.614	1	9.614	6.45	0.0441
AA	33.32	1	33.32	22.34	0.0032
Lack of Fit	2.122	5	0.4245	0.28	0.9054
Pure Error	8.947	6	1.491		
Total (Corrected)	93.38	14			

Table S2. Analysis of variance for TPC (mg GAE/g dm) of Matico

Source	*Sum of Squares*	*DF*	*Mean Square*	*F-Value*	*P-Value*
A:Methanol concentration (%)	25.06	1	25.06	214.90	0.0000
B:Time (min)	0.4232	1	0.4232	3.63	0.1055
AA	0.7088	1	0.7088	6.08	0.0488
AB	0.7656	1	0.7656	6.56	0.0428
Lack of Fit	0.1184	4	0.02959	0.25	0.8973
Pure Error	0.6998	6	0.1166		
Total (Corrected)	27.78	14			

Table S3. Analysis of variance for ABTS (μmol TE/g dm) of Matico

Source	*Sum of Squares*	*DF*	*Mean Square*	*F-Value*	*P-Value*
A:Methanol concentration (%)	3830.	1	3830.	249.41	0.0000
B:Time (min)	3.138	1	3.138	0.20	0.6672
AA	537.2	1	537.2	34.98	0.0010
AB	52.85	1	52.85	3.44	0.1130
Lack of Fit	70.83	4	17.71	1.15	0.4164
Pure Error	92.15	6	15.36		
Total (Corrected)	4586.	14			

Table S4. Analysis of variance for DPPH (μmol TE/g dm) of Matico

Source	*Sum of Squares*	*DF*	*Mean Square*	*F-Value*	*P-Value*
A:Methanol concentration (%)	2975.68	1	2975.68	212.04	0.0000
B:Time (min)	84.2402	1	84.2402	6.00	0.0498
AA	94.0748	1	94.0748	6.70	0.0413
Lack of Fit	125.582	5	25.1164	1.79	0.2491
Pure Error	84.2029	6	14.0338		
Total (Corrected)	3363.78	14			

ANOVA – Box-Behnken analysis for the experimental Chacruna data (Non-reparametrized model)

Table S5. Analysis of variance for TEY (%) of Chacruna

Source	*Sum of Squares*	*DF*	*Mean Square*	*F-Value*	*P-Value*
A:Methanol concentration (%)	0.00005	1	0.00005	0.00	0.9947
B:Time (min)	2.54251	1	2.54251	2.86	0.2326
C:Power (W)	10.2378	1	10.2378	11.53	0.0768
AA	24.2294	1	24.2294	27.30	0.0347
AB	0.511225	1	0.511225	0.58	0.5272
AC	10.0806	1	10.0806	11.36	0.0779
BB	7.61213	1	7.61213	8.58	0.0995
BC	0.1444	1	0.1444	0.16	0.7257
CC	28.3137	1	28.3137	31.90	0.0299
Lack of Fit	24.8387	3	8.27957	9.33	0.0984
Pure Error	1.77527	2	0.887633		
Total (Corrected)	111.253	14			

Table S6. Analysis of variance for TPC (mg GAE/g dm) of Chacruna

Source	*Sum of Squares*	*DF*	*Mean Square*	*F-Value*	*P-Value*
A:Methanol concentration (%)	127.6	1	127.6	55.54	0.0175
B:Time (min)	55.8096	1	55.8096	24.29	0.0388
C:Power (W)	9.11645	1	9.11645	3.97	0.1846
AA	49.8104	1	49.8104	21.68	0.0432
AB	10.0172	1	10.0172	4.36	0.1720
AC	19.6249	1	19.6249	8.54	0.0998
BB	66.4441	1	66.4441	28.92	0.0329
BC	0.01	1	0.01	0.00	0.9534
CC	129.42	1	129.42	56.33	0.0173
Lack of Fit	98.5637	3	32.8546	14.30	0.0661
Pure Error	4.59527	2	2.29763		
Total (Corrected)	583.849	14			

Table S7. Analysis of variance for ABTS (μmol TE/g dm) of Chacruna

Source	*Sum of Squares*	*DF*	*Mean Square*	*F-Value*	*P-Value*
A:Methanol concentration (%)	42125.4	1	42125.4	336.19	0.0030
B:Time (min)	25173.2	1	25173.2	200.90	0.0049
C:Power (W)	1576.41	1	1576.41	12.58	0.0711
AA	833.402	1	833.402	6.65	0.1232
AB	13694.9	1	13694.9	109.29	0.0090
AC	4363.26	1	4363.26	34.82	0.0275
BB	21003.2	1	21003.2	167.62	0.0059
BC	22.2312	1	22.2312	0.18	0.7145
CC	34775.9	1	34775.9	277.54	0.0036
Lack of Fit	55236.7	3	18412.2	146.94	0.0068
Pure Error	250.605	2	125.302		
Total (Corrected)	203415.	14			

Table S8. Analysis of variance for DPPH (µmol TE/g dm) of Chacruna

Source	*Sum of Squares*	*DF*	*Mean Square*	*F-Value*	*P-Value*
A:Methanol concentration (%)	20039.0	1	20039.0	6.13	0.1317
B:Time (min)	5842.8	1	5842.8	1.79	0.3131
C:Power (W)	4809.35	1	4809.35	1.47	0.3491
AA	9806.56	1	9806.56	3.00	0.2255
AB	1448.94	1	1448.94	0.44	0.5741
AC	5987.66	1	5987.66	1.83	0.3087
BB	5249.7	1	5249.7	1.61	0.3327
BC	1994.96	1	1994.96	0.61	0.5165
CC	8227.9	1	8227.9	2.52	0.2536
Lack of Fit	9508.19	3	3169.4	0.97	0.5440
Pure Error	6540.55	2	3270.27		
Total (Corrected)	80402.6	14			
